# Occurrence and Determination of Antimicrobial Resistant *Escherichia coli* Isolates in Fish and Vegetables as Indicator Organism of Faecal Contamination in Dar es Salaam, Tanzania

**DOI:** 10.1155/2021/6633488

**Published:** 2021-02-16

**Authors:** Francis Mwanza, Erick Vitus Gabriel Komba, Dominic Mukama Kambarage

**Affiliations:** ^1^Department of Veterinary Medicine and Public Health, College of Veterinary Medicine and Biomedical Sciences, Sokoine University of Agriculture (SUA), P.O. Box 3015, Morogoro, Tanzania; ^2^Ministry of Fisheries and Livestock, P.O. Box 86, Chongwe, Lusaka, Zambia; ^3^Mwalimu Julius Kambarage Nyerere University of Agriculture and Technology, P.O. Box 976, Musoma, Mara (HQ-Butiama), Tanzania

## Abstract

*Escherichia coli* such as *E. coli* O157:H7, a non-sorbitol-fermenting (NSF) *E. coli*, is an essential human pathogen among other common zoonotic pathogens carried by animals especially cattle. They are discharged through cattle faeces into the environment. With the increasing practice of urban farming, livestock manure is used as organic fertiliser in either fish ponds or vegetable gardens. This practice increases the risk of transmission of such pathogens to humans. This study aimed at determining the occurrence, antimicrobial resistance profiles, and genetic relatedness of *E. coli* isolates from manure, vegetables, and fish. Microbiological standard methods were used to isolate and identify *E. coli* isolates from manure, vegetable, and fish samples. Confirmed isolates on biochemical tests were tested for resistance against six antibiotics using the disc diffusion method. Enterobacterial repetitive intergenic consensus polymerase chain reaction (ERIC-PCR) typing method was used to generate fingerprints and determine the genetic relatedness of the *E. coli* isolates. Of 156 samples including 89 manure, 53 vegetables, and 16 fish, 36 (23.1%) samples were positive for *E. coli* from where a total of 48 *E. coli* different isolates were recovered that were subjected to antimicrobial susceptibility testing and genetic relatedness. Of these isolates, 25 (52.1%) were resistant to at least one antimicrobial agent and 12 (48.0%) showed multidrug resistance. ERIC-PCR profiles of *E. coli* isolates from manure, vegetables, and fish showed genetic diversity with genetic relatedness ranging from 74.5% to 100%. Nine phylogenetic clusters (I–IX) determined at 90% threshold level of genetic relatedness were identified among the isolates. This study determined the occurrence, antimicrobial resistant patterns, and genetic diversity of antimicrobial-resistant *E. coli* isolates from different sources. This study showed the potential of microbial health risk to humans through contamination, and hence, it is necessary to monitor and improve husbandry practices in urban farming.

## 1. Introduction

*Escherichia coli* strains including the Shiga toxin-producing *E*. *coli* (STEC) O157:H7 are of worldwide public health importance and are associated with haemolytic uremic syndrome (HUS) and enteric/diarrheal diseases in humans especially children [1–5]. Majority of these STEC strains are non-sorbitol-fermenting (NSF) on sorbitol MacConkey (SMAC) agar. This characteristic forms the basis for the identification of these strains as they cannot be differentiated from commensal *E. coli* on selective media except for some serotypes of STEC. Non-sorbitol-fermenting *E. coli* is an essential zoonotic pathogen in humans carried by animals especially cattle and is discharged through cattle faeces into the environment. Livestock including cattle and poultry contribute about 85% of animal faecal waste to the environment worldwide, a contribution less than that of the human population [6]. Out of the total solid waste produced in most African largest cities, only 20–50% is collected by the local authorities for proper disposal [7]. The uncollected animal faecal waste results in increased accumulation of livestock manure in urban areas and poses a health risk to humans. The risk is even heightened with the use of both liquid and solid manure contaminated with zoonotic pathogens including *E. coli* in vegetable or fish farming [8].

In developing nations including Tanzania, rapid population growth and urbanization in cities have resulted in the adoption of urban farming practices as a livelihood strategy among people in urban and periurban communities [9]. The manure deposited in the fish ponds acts as fertiliser that supports the growth of photosynthetic organisms on which the fish feeds [10]. During fish harvesting, the water from the pond is also used to water vegetable gardens and the vegetables produced can be consumed by people as well as livestock. This type of urban integrated farming helps the farmer to reduce costs of production and maximize profits in an urban setup where there is limited land space. Urban farming involves livestock that is kept within residential areas due to limited land; however, animal husbandry practices including the use of antimicrobial agents if not properly managed and monitored may have a residual effect on livestock products including manure. The irrational use of antimicrobial agents especially those intended for humans in livestock or fish can lead to the development of antimicrobial resistant organisms [11]. The increased prevalence of antimicrobial resistant organisms in food animals and animal products including livestock, poultry, and fish promotes transmission to humans through the food chain [12]. The risk can be increased if zoonotic pathogens are involved and are allowed to contaminate the water or food meant for human consumption. This contamination of water or food with zoonotic enteric pathogens including *E. coli* may not only cause infections such as haemolytic uremic syndrome (HUS) [13] but can also lead to transmission of antimicrobial resistant *E. coli* [14].

This study, therefore, aims to determine the occurrence of potential pathogens using NSF *E. coli* as an indicator organism. The demonstration of its antimicrobial resistance and genetic relatedness of the isolates from manure, vegetable, and fish grown in an integrated system show the potential health risk urban farming may pose.

## 2. Materials and Methods

### 2.1. Study Area

**Figure 1:**
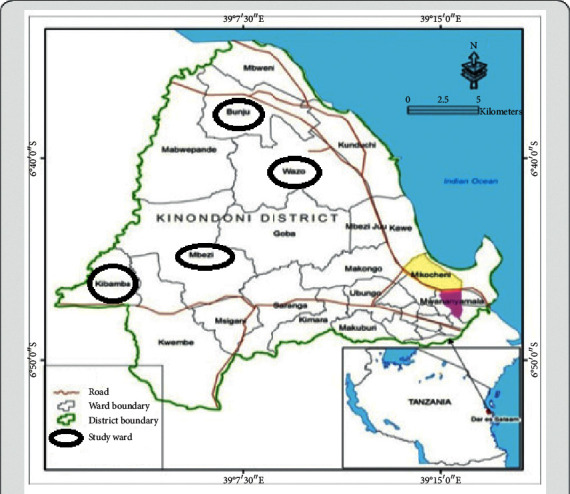
Map showing Kinondoni Municipality, location, and names of wards included in the study. Source: USDM IRA-GIS Unit (2015).

The study was conducted in Kinondoni Municipality, the Dar es Salaam region of Tanzania, located on the western coast of the Indian Ocean. The municipality has the highest number of people, approximately 1,775,049, covering an area of 522.3 square kilometres consisting of four divisions namely Kinondoni, Magomeni, Kibamba, and Kawe. The study was conducted in two of the four divisions of the municipality including Bunju and Wazo wards in the Kawe division and Mbezi and Kibamba wards in the Kibamba division where urban farming including the use of livestock manure as fertiliser is practised (Figure 1). The sampling period was from 3^rd^ March 2016 to 9^th^ April 2016 from a total of 93 randomly selected households that practised at least more than one farming activity including livestock keeping, fishing, or vegetable gardening. Due to constraints of not finding all the three farming activities present at each household, only 156 samples were collected from the households including 89 manure, 51 vegetables, and 16 fish samples for determination of the occurrence of NSF *E. coli* from the different sources.

### 2.2. Sample Collection and Preparation

Approximately 100 g of fresh faecal samples for cattle and chickens were collected using a sterile examination glove and placed in a cooler box containing ice packs. The fresh faecal samples were collected at least within five minutes after being deposited on the ground in cases where direct collection per rectum proved difficult. Three to five faecal deposits were collected and pooled to make one household sample for the fresh manure. Dry faecal samples were also collected from the areas where they were deposited ready for use in the vegetable garden or sale.

Similarly, portions of dry manure were collected from three to five sites within the deposition area to make a pooled faecal sample. Fish was collected from the fish ponds by use of fishing and scoop nets. The samples were stored in a sealable plastic bag and transported in a cooler box with ice packs and stored at 4–8°C before being analyzed. The edible parts of the leafy vegetables such as *African spinach* (*mchicha*) that was ready to be harvested for sale or home consumption were randomly collected from the household gardens. Approximately one household vegetable sample consisted of three to five edible parts of the leafy vegetables collected from the household garden. After collection, all the samples were transported in a cooler box containing ice packs to the Public Health Laboratory at SUA in Morogoro and were analyzed within 48 hours after collection.

Standard methods were used to prepare samples for NSF *E. coli* isolation where 1 g of manure or intestinal contents for fish was added to 4 mL of Maximum Recovery Diluent (MRD) (Oxoid Ltd., Basingstoke, Hampshire, UK) and incubated at 37°C for 24 h after homogenization for one minute. The vegetable samples were manually cut into small pieces using a sterile blade, and 10 g was measured and placed in a stomacher bag. Then, 90 mL of MRD solution was added to the sample, and the mixture was stomached for 1 minute at low speed. The suspension was transferred into a clean bijou bottle for *E. coli* isolation.

### 2.3. *E. coli* Isolation

One loop full (approximately 10 *μ*L) of the prepared sample suspension was collected and streaked on to MacConkey agar (Oxoid Ltd., Basingstoke, Hampshire, UK) to obtain a primary culture. This was followed by incubation for 24 h at 37°C. From the primary culture, an average of four pure typical and atypical *E. coli* colonies were subcultured on to Sorbitol-MacConkey agar (Oxoid Ltd., Hampshire, UK) and incubated at 37°C for 24 hours in order to obtain independent NSF *E. coli* colonies. All the independent NSF *E. coli* colonies were subjected to biochemical tests screening. The indole, methyl-red, Voges Proskauer, and citrate (IMViC) (Sigma-Aldrich Co., Switzerland) tests were used for the confirmation of *E. coli* isolates according to the manufacturer's instructions. Isolates that were indole-positive, methyl-red-positive, Voges Proskauer-negative, and citrate-negative were confirmed to be *E. coli.* The confirmed NSF *E. coli* isolates on biochemical tests were stored in Mueller Hinton Broth containing 15% v/v glycerol at −20°C for antimicrobial susceptibility testing and molecular analyses to determine the genetic relatedness. Since only single pure colonies were picked, the recovered *E. coli* isolates even from the same sample were treated as independent strains during antimicrobial resistance testing and genetic relatedness determination.

### 2.4. Antimicrobial Susceptibility Testing

The antimicrobial resistance testing of all NSF *E. coli* isolates was performed using the disc diffusion method as described by the Clinical and Laboratory Standards Institute [15]. The pure culture of NSF *E. coli* colony was suspended into the test tube containing 10 mL of 0.9% sodium chloride solution, and its concentration (turbidity) was compared to the standard 0.5 McFarland (Oxoid Ltd., Hampshire, UK). The sterile swab was used to inoculate the bacterial suspension by completely streaking onto the previously prepared Mueller Hinton agar (Oxoid Ltd, Hampshire, UK) plates. After placing the antimicrobial susceptibility test discs (Oxoid Ltd., Hampshire, UK), the agar plates were incubated overnight at 37°C, and the inhibition zone diameters produced around the antimicrobial discs were measured using a ruler and interpreted using the Kirby-Bauer chart. Six antimicrobial agents commonly used in both livestock and humans were used to test for resistance including amoxicillin (AML-10 *μ*g), tetracycline (TE-10 *μ*g), azithromycin (AZM-15 *μ*g), ampicillin (AMP-25 *μ*g), ceftriaxone (CRO-30 *μ*g), and ciprofloxacin (CIP-1 *μ*g).

### 2.5. DNA Extraction

Genomic *E. coli* DNA was extracted by the heat lysis method after growing all isolates on nutrient broth overnight at 37°C and checking them for purity on nutrient agar (Thermo Scientific, UK) to obtain fresh cultures as described by Englen and Kelley [16]. Approximately 3–5 colonies were picked and suspended in 100 *μ*L of sterile water in an Eppendorf tube to obtain a turbid suspension of bacterial cells. By using a Techne's Dri-Block® heater (Bibby-Scientific Ltd., Staffordshire, UK), the bacterial suspension was heated at 95°C for 15 minutes. The suspension was centrifuged at 12,000 ×g for 10 minutes to obtain the supernatant and stored until needed at −20°C. Only 5 *μ*L was directly used as DNA template for PCR.

### 2.6. DNA Amplification by ERIC-PCR

The amplification of genomic DNA was carried out in a 25 *μ*L total volume containing 3 *μ*L of deionised water, 3.5 *μ*L of each forward primer ERIC-1R, 5′-ATG TAA GCT CCT GGG GAT TCA C-3′ and reverse primer ERIC-2, 5′-AAG TAA GTG ACT GGG GTG AGC G-3′ [15], 10 *μ*L of master mix, and 5 *μ*L of the DNA template. The reactions were conducted using a Takara PCR Thermal cycler (Takara Bio, Japan). The following thermocycling conditions were used; an initial denaturation temperature of 94°C for 7 minutes followed by 34 cycles consisting of denaturation at 94°C for 30 seconds, annealing at 38°C for 1 minute, extension at 72°C for 5 minutes, and a single final extension step at 72°C for 15 minutes before holding temperature at 4°C.

### 2.7. Gel Electrophoresis

The amplicons were electrophoresed on a 1.5% Agarose gel in 100 mL solution of 1 × TBE (Tris-Borate-EDTA) buffer, along with 100bp DNA molecular weight markers (Fisher Scientific, UK), and 2 *μ*L of 6x Blue loading dye stained in ethidium bromide (0.5 mg/mL). An electric field of 100V was applied for 45 minutes during electrophoresis before visualizing the amplicons under UV light.

### 2.8. Determination of Genetic Relatedness of *E. coli* Isolates

Cluster analysis of ERIC-PCR products was conducted using BioNumerics software, GelCompar II, version 6.6.11 (Applied Maths, Sint-Martens-Latem, Belgium) to determine the genetic relatedness of *E. coli* isolates from different sources. Using the number of different band method, a dendrogram was generated with optimization and tolerance coefficients both set at 1%. Similarity indices indicated as percentages of the DNA banding patterns on a similarity matrix were generated using the GelCompar software. Since ERIC-PCR was used to amplify conserved regions of DNA, analysis of *E. coli* isolates from different sources, geographical locations, households, and sampling dates was carried out to assess possible cross-contamination based on the similarities of fingerprints.

## 3. Results

NSF *E*. *coli* was isolated in 36 (23.1%: 95% CI; 16.7–30.5) out of the total number of 156 samples collected and yielded 48 different isolates that were subjected to antimicrobial resistance testing and determination of genetic relatedness. Seventeen isolates from both manure (19.1%) and vegetables (33.3%) yielded NSF *E. coli*, while two (12.5%) isolates were from fish (Table 1).

### 3.1. Antimicrobial Resistance Profiles of Obtained Isolates from Different Sources

Out of the 48 *E. coli* isolates comprising 20 manure, 25 vegetables, and three fish subjected to antimicrobial resistance testing, 25 (52.1%) showed resistance to at least one antimicrobial agent including 11 manure, 13 vegetables, and one fish. Resistance was more common to amoxicillin (35.4%), while Ciprofloxacin (2.1%) had the least number of resistant isolates (Table 2).

Out of the total number of isolates that were resistant to at least one antimicrobial agent, 12 (48.0%) displayed multidrug resistance (MDR). Multidrug resistance was considered as the resistance of an isolate to two or more classes of antimicrobial agents [17]. Isolates 14M and 21V from manure and vegetables, respectively, exhibited a similar MDR pattern; however, none of the isolates from fish exhibited MDR (Table 3).

**Figure 2:**
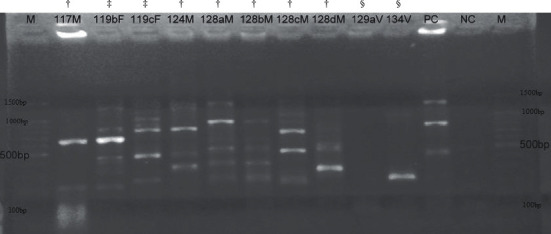
Representative DNA fingerprints from ERIC-PCR of *E. coli* isolates from manure, vegetable, and fish samples identified as follows: ^†^, isolates from manure; ^§^, isolates from vegetable; ^‡^, isolates from fish; PC, positive control (*E. coli* K-12); NC, negative controls; M, 100 base pairs (bp) DNA ladder.

The ERIC-PCR profiles produced after gel electrophoresis revealed highly polymorphic DNA fragments both in number and size among the isolates. This variation was demonstrated even between isolates from the same sample type and source (Figure 2). All isolates that were subjected to ERIC-PCR produced a range of 1 to 9 bands with an average of 4 bands per isolate. The proportion of the isolates with a particular band size among the sources ranged from approximately 100bp (17.0%, 8/47) to 1,700bp (2.1%, 1/47). The most common band size shared among the isolates was 300bp (61.7%, 29/47) (Figure 2).

### 3.2. Genetic Relatedness among the Antimicrobial-Resistant *E. coli* Isolates

**Figure 3:**
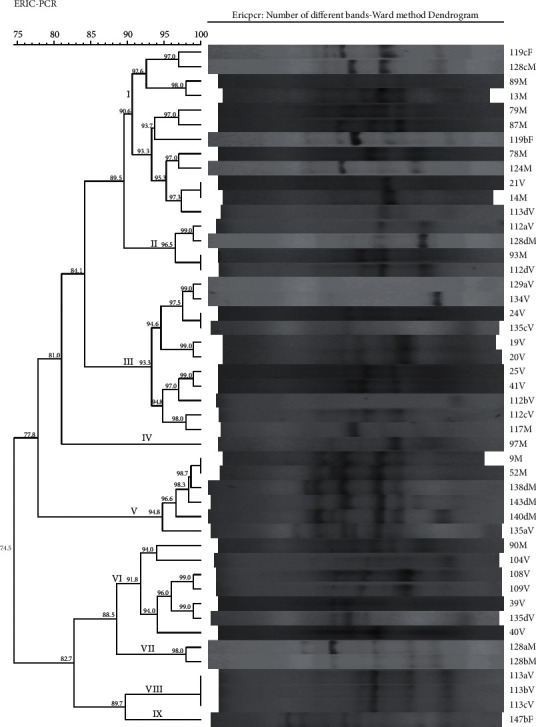
Dendrogram showing ERIC-PCR band patterns, clusters, and genetic similarity of *E. coli* isolates from different sources identified as follows: M, isolate from manure; V, isolate from vegetable; F, isolate from fish; a, b, c, d, multiple isolates from a sample; (I–IX), identified clusters.

ERIC DNA fingerprints were used to construct a phylogenetic tree to demonstrate the genetic similarity among the isolates from different sources. Nine clusters were identified and designated as (I) to (IX) for identification purposes. The similarity level among isolates ranged from 74.5% to 100%. Clusters were identified based on a similarity threshold of 90% and above. Cluster (I) was the largest, while clusters (IV) and (IX) were the smallest in size with only one isolate making a cluster for each (Figure 3). Isolates from the same household which included 117M from manure, 112V from vegetable with subisolates 112aV, 112bV, 112cV, and 112dV, and also 119F from fish with subisolates 119bF and 119cF exhibited different antimicrobial resistant patterns and were grouped in different clusters. Isolate 112aV produced a MDR pattern with 112dV resistant only to TE and both were in cluster (II) with a genetic similarity level of 96.5%. Isolate 112dV from vegetables showed 100% similarity with isolate 93M from manure. Isolate 117M from manure was more similar to subisolates 112cV (98.0%) and 112bV (94.8%) from the vegetable in cluster (III) with no MDR pattern observed; however, isolates 14M and 21V from manure and vegetable, respectively, in cluster (I) showed a 100% genetic similarity. This variation in genetic relatedness and resistant patterns of isolates was demonstrated between households, sample types, and sources as well as ward locations from where the samples were collected (Table 4).

## 4. Discussion

### 4.1. Prevalence of NSF *E. coli*

A number of studies have demonstrated the potential for spread of zoonotic pathogens including pathogenic *E. coli* O157:H7 from livestock to humans through water- and food-borne sources [4, 18–20], especially where integrated urban farming is being practised. This study, however, revealed a significantly higher prevalence 23.1%, *p*=0.0031 than the 13.7% reported by Lupindu et al. [4] from a similar study in urban and periurban areas of the Morogoro region of Tanzania. The observed higher prevalence reported in this study could be due to the different sample sources and methodology with the previous study. This study also involved the sampling of leafy vegetables, which was not part of the previous study; however, the small volume of the sample used in this study especially for vegetables would have affected the test sensitivity, resulting in the isolation of low numbers of NSF *E. coli*.

Different studies have reported occurrence and antimicrobial resistance among NSF *E. coli* from livestock manure, vegetables, and fish [4, 21–23]. The levels of resistance for the isolates to selected antimicrobial agents in this study were not very different from those obtained in other studies within Tanzania and other regions worldwide. Other studies have also reported lowest antimicrobial resistance rates for ciprofloxacin among pathogenic *E.coli* isolates for as low as 0.0% [22, 24, 25] similar to the finding in this study.

It was noted in the present study that isolates from vegetables had a similar MDR pattern to isolates from manure. Of particular interest, isolates 14M and 21V from manure and vegetables, respectively, exhibited similar MDR, a finding similar to what was reported by Holvoet et al. [25]. This finding demonstrates a possible common origin of isolates despite being isolated from different sources. The common origin of zoonotic pathogens including *E. coli* can also result in the sharing of genes that confer antimicrobial resistance ability; hence, it is a public health risk to humans [26]. The MDR reported in this study is of great public health concern especially with resistance observed in cephalosporin (ceftriaxone) and quinolone (ciprofloxacin) groups of antibiotics. This finding indicates the likelihood of the presence of *E. coli* strains that produce extended-spectrum *β*-lactamases (ESBLs) that are being reported in increased frequency in many countries and would need further research to elucidate the health risk they pose in urban farming. Furthermore, fish have also been reported to be an essential medium through which MDR including that of ESBLs *E. coli* producing strains develops and can be transmitted to humans through consumption thereby posing a heath risk [11].

### 4.2. Genetic Relatedness of NSF *E. coli* Isolates from Different Sources

There are many studies [1, 27, 28] previously conducted to determine genetic relatedness of *E. coli* isolates by using ERIC-PCR, which is one of the PCR-based typing methods used to determine the genetic similarities of different strains of Enterobacteriaceae including *E. coli* O157:H7 originating from different sources. Alternatively, other researchers have used more superior methods including pulse-field gel-electrophoresis (PFGE), which is a standard gold method of achieving this objective [21, 29]. In this study, ERIC-PCR was conducted in order to get information on the genetic diversity of antimicrobial resistant *E. coli* from different sources including manure, vegetables, and fish. The data obtained would be used to determine possible cross-contamination from one source to the other based on genetic similarities of fingerprints of the isolates.

ERIC-PCR successfully discriminated *E. coli* strains of isolates from manure, vegetables, and fish into clusters based on their ERIC-PCR DNA profiles. Nine clusters were identified with the overall similarity ranging from 74.5% to 100% (Figure 3). This result shows increased discriminatory power of *E. coli* strains by ERIC-PCR among isolates than that previously reported by Ateba and Mbewe [1] who used a similar method and found that, out of 94 of *E. coli* O157:H7 isolates, 8 clusters were identified with the overall similarity ranging from 71% to 91%; however, the result of this study is in agreement with what was reported by Lupindu et al. [21] despite the use of PFGE method that yielded isolates with an overall similarity of 53.3% to 100% with 8 clusters identified. Therefore, these findings also confirm the discriminatory power of bacterial clones by use of the ERIC-PCR method to be equivalent to that of PFGE as reported by Ibenyassine et al. [27].

Some clusters contained isolates that exhibited 100% genetic similarity and displayed similar MDR patterns despite having been isolated from different sources. An example of such was cluster (I) where isolate 21V from vegetables and 14M from manure had a similar MDR pattern of AML, TE, and AMP. This may suggest possible contamination of *E. coli* between the two integrated farming components, although there is a lack of concrete conclusion on the direction of flow as reported by Lupindu et al. [21]. Another interesting result in this study is that isolates 9M from Wazo and 52M from Bunju showed 100% similarity, while isolates 24V from Wazo and 135cV from Kibamba also showed 100% similarity. In addition, isolates 24V and 9M, despite not being so similar and were both collected from Wazo at different households and dates, showed a similar MDR pattern; however, some isolates even from different sources showed 100% genetic relatedness. The finding of isolates from the same household and clusters including cluster (II), (III), and (VIII) is in agreement with a study by Ateba and Mbewe [1] that indicated that strains of the same subtype would lie within the 95% and 100% of genetic similarity interval. This is the case regardless of the DNA band optimization and tolerance settings, which were both set at 1% in this study during dendrogram formation.

## 5. Conclusions

This study determined the presence, antimicrobial resistant patterns, and genetic diversity of *E. coli* isolates as an indicator organism in livestock manure, vegetables, and fish. The public health risk posed is heightened by the contamination of antimicrobial resistant and genetically diverse *E. coli* between different components of the farming systems through husbandry practices. This study also confirms the ERIC-PCR method to be an alternative and cost-effective method that can be used to separate bacterial isolates from different sources and determine their genetic relatedness.

Therefore, there is need to sensitize communities to raise awareness on the health risks of urban farming and advocate for the promotion of good husbandry practices in urban farming. Also, recognizing the antimicrobial resistance, current findings in this study, there is an apparent need to develop national plans to monitor and regulate the use of chemicals and antimicrobials used in the country. We recommend that further molecular studies should be conducted that would identify and characterize common circulating zoonotic pathogens including *E. coli* in livestock, humans, vegetables, and fish to determine the species, their associated risk factors of flow, survival, and transmission in urban farming. Serological tests confirming the serotypes and tests for known virulence genes via PCR that was not performed in this study should be further investigated to determine whether the isolates are potential human pathogens or not.

## Figures and Tables

**Table 1:** Distribution of proportions for NSF *E. coli* from different sources (*n* = 156).

Source	Number of samples	NSF *E. coli* (%)
Manure	89	17 (19.1%)
Vegetables	51	17 (33.3%)
Fish	16	2 (12.5%)

Total (overall)	156	36 (23.1%)

**Table 2:** Proportions of antimicrobial resistant *E. coli* isolates from different sources.

	Sample source		
Antibiotic (concentration)	Manure (%)	Vegetable (%)	Fish (%)
Amoxicillin (10 *μ*g), AML	5 (25.0%)	11 (44.0%)	1(33.3%)
Tetracycline (10 *μ*g), TE	10 (50.0%)	3 (12.0%)	0 (0.0%)
Ampicillin (25 *μ*g), AMP	3 (15.0%)	9 (36.0%)	0 (0.0%)
Azithromycin (15 *μ*g), AZM	4 (20.0%)	7 (28.0%)	0 (0.0%)
Ceftriaxone (30 *μ*g), CRO	3 (15.0%)	3 (12.0%)	0 (0.0%)
Ciprofloxacin (1 *μ*g), CIP	1 (5.0%)	0 (0.0%)	0 (0.0%)

**Table 3:** Multidrug resistance patterns of NSF *E. coli* isolates.

Isolate ID	Source	Antimicrobial resistant pattern
143M	Manure	$\left.\begin{array}{c} \text{AML, TE, AZM}\\ \text{AML, TE, AZM}\\ \text{AML, TE, AZM} \end{array}\right\}1$
24V	Vegetable	
9M	Manure	
14M	Manure	$\left.\begin{array}{c} \text{AML, TE, AMP}\\ \text{AML, TE, AMP}\\ \text{AML, TE, AMP} \end{array}\right\}2$
21V	Vegetable	
78M	Manure	
25V	Vegetable	$\left.\begin{array}{c} \text{AML, AZM, AMP}\\ \text{AML, AZM, AMP}\\ \text{AML, AZM, AMP}\\ \text{AML, AZM, AMP}\\ \text{AML, AZM, AMP} \end{array}\right\}3$
108V	Vegetable	
112aV	Vegetable	
113aV	Vegetable	
113cV	Vegetable	
128aM	Manure	$\left.\begin{array}{c} \text{TE},\text{ AZM},\text{ CRO} \end{array}\right\}4$

**Table 4:** Table showing isolate sources including ward, household ID, and date the sample was collected.

Isolate	Source	Ward name	HH_ID	Sample date
119cF	Fish	Mbezi	21	04/04/2016
128cM	Cattle manure	Kibamba	11	04/04/2016
89M	Poultry manure	Mbezi	23	29/03/2016
13M	Poultry manure	Wazo	24	03/03/2016
79M	Cattle manure	Mbezi	20	29/03/2016
87M	Cattle manure	Mbezi	5	29/03/2016
119bF	Fish	Mbezi	21	04/04/2016
78M	Poultry manure	Bunju	8	16/03/2016
124M	Cattle manure	Kibamba	12	04/04/2016
21V	Vegetable	Wazo	20	03/03/2016
14M	Poultry manure	Wazo	7	03/03/2016
113dV	Vegetable	Mbezi	8	04/04/2016
112aV	Vegetable	Mbezi	21	04/04/2016
128dM	Cattle manure	Kibamba	11	04/04/2016
93M	Cattle manure	Mbezi	20	29/03/2016
112dV	Vegetable	Mbezi	21	04/04/2016
129aV	Vegetable	Kibamba	12	04/04/2016
134V	Vegetable	Kibamba	14	04/04/2016
24V	Vegetable	Wazo	21	03/03/2016
135cV	Vegetable	Kibamba	21	04/04/2016
19V	Vegetable	Wazo	1	03/03/2016
20V	Vegetable	Wazo	23	03/03/2016
25V	Vegetable	Wazo	2	03/03/2016
41V	Vegetable	Wazo	15	07/03/2016
112bV	Vegetable	Mbezi	21	04/04/2016
112cV	Vegetable	Mbezi	21	04/04/2016
117M	Poultry manure	Mbezi	21	04/04/2016
97M	Cattle manure	Mbezi	22	29/03/2016
9M	Cattle manure	Wazo	22	03/03/2016
52M	Cattle manure	Bunju	1	11/03/2016
138dM	Cattle manure	Kibamba	7	09/04/2016
143dM	Cattle manure	Kibamba	5	09/04/2016
140dM	Cattle manure	Kibamba	4	09/04/2016
135aV	Vegetable	Kibamba	21	04/04/2016
90M	Cattle manure	Mbezi	16	29/03/2016
104V	Vegetable	Mbezi	11	29/03/2016
108V	Vegetable	Mbezi	2	29/03/2016
109V	Vegetable	Mbezi	7	29/03/2016
39V	Vegetable	Wazo	9	07/03/2016
135dV	Vegetable	Kibamba	21	04/04/2016
40V	Vegetable	Wazo	14	07/03/2016
128aM	Cattle manure	Kibamba	11	04/04/2016
128bM	Cattle manure	Kibamba	11	04/04/2016
113aV	Vegetable	Mbezi	8	04/04/2016
113bV	Vegetable	Mbezi	8	04/04/2016
113cV	Vegetable	Mbezi	8	04/04/2016
147bF	Fish	Kibamba	3	09/04/2016

## Data Availability

The data used to support the results of this study are available from the corresponding author upon request.
